# The hierarchical framework of wellbeing (HiFWB)

**DOI:** 10.3389/fpsyg.2025.1515423

**Published:** 2025-02-28

**Authors:** David J. Disabato, Fallon R. Goodman, Todd B. Kashdan

**Affiliations:** ^1^Psychology Department, Baldwin Wallace University, Berea, OH, United States; ^2^Department of Psychological and Brian Sciences, George Washington University, Washington, DC, United States; ^3^Department of Psychology, George Mason University, Fairfax, VA, United States

**Keywords:** wellbeing, happiness, quality of life, measurement, assessment

## Abstract

**Purpose:**

Several decades of research on wellbeing has resulted in a variety of conceptual models used to measure wellbeing. The historical motivations behind these conceptual models have emphasized their differences to the point of clouding the wellbeing measurement landscape. A synthesis of the wellbeing literature is needed to move the field forward and guide future research.

**Methods:**

In this review, we synthesize literature on the measurement of wellbeing from the past 50 years and present The Hierarchical Framework of Wellbeing (HiFWB) that organizes multiple prior models.

**Results:**

We propose a general factor of wellbeing (i.e., “h” factor) at the top level of the hierarchy analogous to “g” in the intelligence literature and “p” in the psychopathology literature. Building off prior conceptualizations, we define general well-being as “the experience of personally valued fulfillment within one's life.” We detail the theoretical rationale and empirical evidence behind four hierarchical levels: general (i.e., “h” factor), lenses (e.g., subjective wellbeing), contents (e.g., affects), and characteristics (e.g., positive affect). Example wellbeing constructs are proposed for each level of HiFWB while emphasizing the hierarchical structure is prioritized above any (arbitrary) list of constructs. We discuss various approaches to distinguishing predictors of wellbeing from wellbeing itself (i.e., preventing tautologies) and how they fit into our framework. Considering the bulk of the empirical evidence comes from Western, Educated, Industrialized, Rich, Democratic (WEIRD) cultures, constraints on generalizability are important. Throughout, we compare and contrast HiFWB to other hierarchical structures in psychological science (e.g., five factor model of personality).

**Conclusion:**

The HiFWB is a flexible, encompassing, evidence-based framework for wellbeing conceptualization and measurement in WEIRD populations.

Wellbeing science sharply increased at the turn of the century with the mobilization of researchers, grant funding, and public interest under the umbrella term “positive psychology” (Seligman and Csikszentmihalyi, 2000). One foundation of a scientific field is measurement, and the value of scientific evidence rests on valid connections between theoretical constructs and their measures. For wellbeing, countless constructs and associated measures have been developed. A review identified a staggering 99 different measures capturing 196 different wellbeing constructs (Linton et al., 2016), with more measures published since. Yet, there is little consensus on how to organize these measurement components in a logical, empirical, and theoretically sound manner. There is a need for a framework that organizes wellbeing measures in a data-driven and coherent manner.

To offer greater organization to wellbeing measurement, we propose a framework that subsumes measurement approaches based on existing psychometric evidence. In the hierarchical framework of wellbeing (HiFWB), wellbeing is conceptualized hierarchically with a single general construct “h” at the top, similar to “g” in the intelligence literature or “p” in the psychopathology literature (Bjørndal et al., 2023). Underneath this general factor of wellbeing are three lower levels— lenses, contents, and characteristics of wellbeing—with increasing specificity as one moves down the hierarchy (Ree et al., 2015). We focus on psychological constructs which can be measured quantitatively to support psychological scientists and clinicians (e.g., therapists) interested in assessing wellbeing. Although there are other epistemologies within psychology (e.g., Alexandrova, 2017), we take a neo-positivist approach to conceptualizing wellbeing emphasizing latent variable psychometric research. We review the history of wellbeing measurement and then outline our hierarchical framework, summarizing empirical support and delineating how to make decisions about wellbeing measurement for studies.

## History of wellbeing measurement

The measurement of wellbeing initially surfaced in psychological science half a century ago. Before then, there were intermittent studies of happiness but not a full body of literature (Hartmann, 1934; Wilson, 1967). Happiness had been studied within medicine (e.g., quality of life) largely as a predictor of physical health and mortality (Larson, 1978). The tides turned in the 1970s when calls rang out to measure and study happiness as an outcome in and of itself (Campbell, 1976). In part to gain credibility within psychological science, researchers began using the term “wellbeing” rather than happiness because it sounded more scientific (E. Diener, personal communication, Feb 7, 2019). The first widely adopted measurement model was Diener's model of subjective wellbeing, which included three dimensions (Diener, 1984): (1) life satisfaction—how satisfied a person is with their current life, (2) positive affect—how frequently a person experiences pleasant emotions, and (3) lack of negative affect—how infrequently a person experiences unpleasant emotions. This model of wellbeing, arguably one of the most influential in psychological science, integrated previous research on avowed happiness and affect balance (Bradburn, 1969; Shin and Johnson, 1978).

Some researchers, however, believed that Diener's subjective wellbeing model omitted several important aspects of happiness written about by theorists (Jahoda, 1958), and that happiness could not be reduced to fleeting affective experiences and generic life satisfaction. To address this gap, subsequent models sought to expand the construct of wellbeing. The second widely adopted measurement model was called psychological wellbeing and included six dimensions (Ryff, 1989): (1) self-acceptance—possessing a positive attitude about oneself, including an acknowledgment and acceptance of both good and bad qualities, (2) positive relations with others—the presence of close, satisfying, trusting relationships with other people, (3) personal growth—a feeling of continual development, where one is changing in ways that reflect an expansion in knowledge and effectiveness, (4) purpose in life—the presence of an overarching aim(s) that directs one's life, (5) environmental mastery—feeling able to effectively manage the situational demands presented in everyday affairs, and (6) autonomy—the ability to act in ways that reflect self-reliance and independence from external influence.

Over the years, researchers proposed additional aspects of wellbeing believed to be missing from scientific discourse. For example, a social wellbeing measurement model was created that included five dimensions focused on people as members of a community rather than isolated individuals (Keyes, 1998): (1) social integration—the sense that one is accepted by and has a sense of belonging in one's community, (2) social acceptance—belief that other people are kind and trustworthy, (3) social contribution—feeling that one's contributions are valued and impactful to society, (4) social coherence—belief that the world is sensible and predictable, and (5) social actualization—belief that society is improving for human beings. Other researchers introduced singular dimensions of wellbeing, such as being engrossed or engaged during activities, where a sense of “flow” is experienced for enduring periods of time (Csikszentmihalyi, 1997). Wellbeing researchers continued to identify and create new constructs purported to be omitted from existing models. As the field strove for inclusivity in wellbeing models, a growing number of constructs—some overlapping, some disparate, some the same construct with different names—populated the literature.

As time went on, some researchers sought to select a subset of existing wellbeing dimensions and package them as a measurement model. For example, PERMA is an acronym where P = positive emotions—feelings of joy, contentment, cheer, etc.; E = engagement—feeling absorbed and interested in life; R = positive relationships—feeling socially integrated, cared about, and supported by others; M = meaning—believing that one's life is valuable and connected to something greater than oneself; and A = accomplishment—making progress toward goals and feeling capable of daily activities (Seligman, 2011). A different measurement model proposed 10 dimensions of wellbeing: competence, emotional stability, engagement, meaning, optimism, positive emotion, positive relationships, resilience, self-esteem, and vitality (Huppert and So, 2013). Several other researchers have proposed their own measurement models (Diener et al., 2009; Longo et al., 2017a; Su et al., 2014). There now exist a wide variety of measurement models with their own unique combinations of wellbeing constructs.

With the growing number of wellbeing measurement models, researchers have attempted to synthesis them together into hierarchical models. One of the first we are aware of was Gallagher et al. (2009) who combined the subjective, psychological, and social wellbeing measurement models together into a single hierarchical model. Their hierarchy had a broad level for types of wellbeing and a specific level for the various components. However, this hierarchical model didn't explicitly provide an overarching general wellbeing level. Other hierarchical models have proposed a general wellbeing level that suggests one overarching construct (Diener et al., 2009; Huppert and So, 2013; Khumalo et al., 2010; Longo et al., 2017a; Su et al., 2014), but they do not reconsider a level in between types and components of wellbeing. Another hierarchical model attempts to define wellbeing at various levels of analysis—physiological, emotional, cognitive, meta-cognitive, developmental, and social-ecological—emphasizing positive balance at each level (Sirgy, 2019). While these models consider different levels of scientific analysis from neurotransmitters to social ostracism, the components of wellbeing are not themselves organized hierarchically. In other words, the hierarchy is across levels of scientific analysis rather than the organization of the wellbeing components. The closest we have seen to a true hierarchical model with at least 3 levels is research from South Africa drawing a comparison to hierarchical models of intelligence and the “g” factor (Khumalo et al., 2011; Wissing and Temane, 2008).

## A hierarchical framework of wellbeing

With a growing number of wellbeing measurement models, the science of wellbeing will benefit from a framework that synthesizes them together—one that is not limited to a subset of wellbeing dimensions and recognizes varied wellbeing measurement models with empirical support. To accomplish this goal, we propose the hierarchical framework of wellbeing (HiFWB) that allows for parsimonious lumping of wellbeing and nuanced splitting of wellbeing depending on the purpose of the research. Our aim is to organize and explain published empirical data in a comprehensive, balanced, and scientifically informed way. Our conceptual analysis follows the “Typology” approach with the goal of *differentiating*—“distinguishing, dimensionalizing, or categorizing extant knowledge of the phenomenon, construct, or theory”—wellbeing (p. 23; Jaakkola, 2020). The wellbeing theories and models used were selected based on their prominence in the field of psychological science, especially the personality, social, and clinical domains. We considered all the components of wellbeing offered in the historical theories reviewed above and re-organized them wholistically, unconstrained by any “type” of wellbeing they were previously associated with. We sought to look at the plethora of wellbeing constructs developed in the last half century with new eyes outside the confines of past conceptualizations.

Our hierarchical framework of wellbeing is similar to other broad psychological constructs that use hierarchical frameworks to theoretically explain the diversity and overlap of various dimensions (e.g., intelligence, psychopathology, personality). At the top is a general construct (e.g., g-factor; p-factor; general factor of personality), followed by multiple lower levels in the hierarchy. The number of constructs and their specificity increases as one goes down the hierarchy, as it does, for example, in models of personality: (Big 5) domains, aspects, facets, and nuances.

Our wellbeing framework is composed of four hierarchical levels. The top-level is *1. general wellbeing*, a single overarching construct (i.e., “h” factor) defined as the experience of personally valued fulfillment within one's life. The next level is *2. lenses*, perspectives by which wellbeing is conceptualized from, followed by *3. content(s)* areas that make up the various wellbeing lenses and organize constructs at face value. The lowest level is *4. characteristics* of wellbeing, defined as discrete, clearly defined components of wellbeing that offer practical value in dissecting human experience. Table 1 illustrates this hierarchical framework populated with a set of examples, which we describe below. We emphasize that these are *examples* and not reified categorizations. There are many ways to parse apart wellbeing constructs within a hierarchical model, and it remains to be seen which level of the hierarchy has the strongest empirical support for predicting useful life outcomes.

**Table 1:** Example constructs organized into the hierarchical framework of wellbeing (HiFWB).

**Level**	**Definition**	**Construct**					
1. General	Experience of personally valued fulfillment within one's life	**Wellbeing**					
2. Lenses	Lenses or perspectives by which wellbeing is conceptualized	**Subjective wellbeing**		**Psychological wellbeing**		**Social wellbeing**	
3. Contents	Content areas that make up the various wellbeing lenses	**Affect**	**Appraisal**	**Meaning-making**	**Self-concept**	**Community**	**Interpersonal relationships**
4. Characteristics	Discrete, clearly defined characteristics of wellbeing that offer practical value in dissecting human experience	Positive affect	Life satisfaction	Meaning in life	Self-esteem	Social integration	Positive relations with others
		(Infrequent) Negative affect	Subjective happiness	Purpose in life	Self-compassion	Social contribution	Relationship quality
				Personal growth	Self-efficacy	Sense of community	Perceived social support
					Locus of control		

The constructs presented are examples and are not meant to be exhaustive.

A hierarchical framework does not give preference to one level over another but rather offers different levels of analysis. Some research questions will pertain to general wellbeing (i.e., “h” factor), while others will pertain to specific characteristics (e.g., positive affect, purpose in life). In this way, research on general wellbeing predicting better physical health (e.g., Stranges et al., 2014) and research exploring the differences between life satisfaction and meaning in life (Tov and Lee, 2016) are both consistent within a hierarchical framework of wellbeing. This is analogous to the scientific study of humans more broadly (e.g., RDoC; Research Domain Criteria; Insel et al., 2010). People can be studied at the level of atoms and molecules (i.e., chemical), cells and bacteria (i.e., biological), electrical impulses and blood flow (e.g., neurological), behaviors and facial expressions (e.g., behavioral), thoughts and feelings (e.g., psychological), or collective groups and communities (e.g., sociological). No level of analysis is “correct,” and research questions should inform how and at which level(s) wellbeing is measured (Kashdan et al., 2015).

### General wellbeing

General wellbeing is an open (or fuzzy) concept, per Meehl's (1986) criteria, and researchers often define wellbeing by a combination of selected characteristics (McDowell, 2010). For example, subjective, psychological, and social wellbeing are all defined by a list of specific characteristics more so than a single overarching conceptual definition. Defining specific characteristics of wellbeing (e.g., life satisfaction), intelligence (e.g., vocabulary), or psychopathology (e.g., panic attacks) is more manageable, but moving to define *general* wellbeing, *general* intelligence, or *general* psychopathology becomes difficult. And yet, there is a shared understanding among scientists of what is broadly meant by general wellbeing, and general constructs tend to explain the most variance in important outcomes (Wiernik et al., 2015). When working with general constructs, conceptual precision is sacrificed for empirical prediction. Nevertheless, building off prior conceptualizations of wellbeing—particularly Diener's writings on subjective wellbeing (Diener, 1984) and more recent work on the experience of being well and doing well (e.g., DeYoung and Tiberius, 2023; Martela and Sheldon, 2019)—we offer a conceptual definition of general wellbeing.

General wellbeing is defined as *the experience of personally valued fulfillment within one's life*. Several components of the structure warrant mention. First, at its core, general wellbeing is about one's life. This includes not only the here and now, but also the story that a person tells about their life (an interpretive narrative; McAdams, 2001). It is indirectly about the self, given that the main character in your life story is none other than you (i.e., self-concept). For example, meaning and purpose in the wellbeing literature is meaning and purpose *in life*, not some abstract, philosophical idea (the meaning *of life*). Second, general wellbeing is about what happens in one's overall life. General wellbeing is a global psychological experience as opposed to something unique to a particular context of one's life. In this way, it is distinct from fulfillment in one's career, relationships, or spirituality, which reflect specific contexts of one's life. Third, general wellbeing can be the result of affective and non-affective, subjective and objective components. The word choice of “personally valued” represents the notion that what contributes to a person's fulfillment will result from what is uniquely important to them due to evolutionary adaptations, biology, temperament, personality, and an idiosyncratic life history (e.g., McAdams and Pals, 2006). Even at the highest level of wellbeing, there will be individual differences that reflect what is of proximal concern to an individual as well as their ultimate concerns. There will be some human universals of what tends to be a desirable end state such as the fulfillment of needs for belonging, competence, and autonomy (e.g., Deci and Ryan, 2000). Fourth, the first word in the definition of general wellbeing is “experience,” allowing for subjective reactions, acknowledging research suggesting that thoughts and feelings tend to be more influential on health, growth, and adaptation than objective indicators. However, we chose experience as a term because this does allow for a person to reference objective characteristics of one's life that are personally weighted as important (e.g., number of friends, the existence of a community contribution or legacy that exists beyond the self; van der Deijl, 2017). With our definition, a person living in poverty within a developing country is not excluded from having high general wellbeing, and a person living as an affluent member of a developed country is not assured of high general wellbeing.

Survey studies with various wellbeing dimensions offer empirical support that general wellbeing is a parsimonious measurement model. Model fit indices from one-factor confirmatory factor analyses (CFA) were reasonably good in clinical (*N* = 472; χ^2^ = 281.20, df = 77, GFI = 0.860, CFI = 0.978, RMSEA = 0.075, SRMR = 0.056), student (*N* = 591; χ^2^ = 2,938.41, df = 846, TLI = 0.974, CFI = 0.975, RMSEA = 0.065, SRMR = 0.065), online (*N* = 517; χ^2^ = 111.97, df = 19, TLI = 0.94, CFI = 0.96, RMSEA = 0.10, SRMR = 0.03), United States community (*N* = 4,043; χ^2^ = 18,189.57, df = 888, TLI = 0.954, CFI = 0.957, RMSEA = 0.070, SRMR = 0.064), European community (*N* = 1,438; χ^2^ = 855.82, df = 75, TLI = 0.85, CFI = 0.88, RMSEA = 0.08, SRMR = 0.06), and international community samples (*N* = 7,617, χ^2^ = 970.85, df = 27, TLI = 0.950, CFI = 0.963, RMSEA = 0.068, SRMR = 0.030; Disabato et al., 2016; Franken et al., 2018; Gallagher et al., 2009; Goodman et al., 2018; Petrillo et al., 2015). In these studies, when multi-factor models (e.g., subjective wellbeing, psychological wellbeing, social wellbeing, PERMA, etc.) were tested, the CFA latent correlations ranged from 0.62 to 0.98, suggesting high overlap among factors and providing support for lumping constructs together. Although splitting up general wellbeing into multiple factors significantly improved model fit in each sample, it was unclear whether the added complexity was always worthwhile relative to a more parsimonious approach. For example, in the largest of these samples (*N* = 7,617), the correlations between the different wellbeing factors and various outcome criteria were very similar (i.e., the average correlation difference = 0.07; Disabato et al., 2016).

Despite adequate model fit, there are aspects of these data not captured by a one-factor model. Researchers using CFA have considered the impact of adding factors in addition to a general wellbeing factor (i.e., bifactor models). The central question guiding these efforts is whether the general wellbeing factor dominates the model or shrinks away in response to including other factors. Several studies using bifactor models found a dominant general factor on which almost all characteristics of wellbeing primarily (Bohnke and Croudace, 2016; Chen et al., 2013, 2006; Jovanovic, 2015; Kokko et al., 2013; Longo et al., 2016, 2017b). These characteristics included subjective happiness, life satisfaction, positive affect, (infrequent) negative affect, self-esteem, self-efficacy, meaning in life, purpose in life, value-congruence, vitality, flow, engagement, (low) depression, (low) psychological distress, optimism, social belonging, and positive relations with others. Across the studies cited above, and a study using a second-order factor model (Kallay and Rus, 2014), total scores from the indicators of general wellbeing showed reliability coefficients ranging from 0.71 to 0.86. The largest of these studies to date (*N* = 7,521) found that 14 different characteristics of wellbeing converged on a general wellbeing factor, with almost all standardized loadings >0.40 (Longo et al., 2017b). After accounting for a general factor, the average residual correlation between wellbeing characteristics ranged from 0.04 to 0.08, suggesting a general factor of wellbeing captured most of the correlations.

While not capturing all aspects of the data, modeling wellbeing as a single, broad factor offers a parsimonious measurement option for researchers and practitioners. Burrowing from the intelligence and psychopathology literatures and consistent with Bjørndal et al. (2023), we label this the “h” factor of happiness.

### Lenses of wellbeing

The second-highest level of the hierarchy contains lenses of wellbeing, defined as various perspectives by which wellbeing can be conceptualized. This has perhaps been the most controversial level, with some people decisively declaring the importance of different wellbeing lenses (Keyes and Annas, 2009; Waterman, 2008), while others question the validity and utility of these distinctions (Biswas-Diener et al., 2009; Kashdan et al., 2008). Studies cited above found correlations as high as 0.98 between “lenses” of wellbeing (Goodman et al., 2018).

Still, not all studies found such high correlations between lenses of wellbeing. When exploratory structural equation modeling (ESEM) is used, evidence for multiple lenses of wellbeing has emerged. ESEM is an exploratory factor analytic model placed within a structural equation modeling framework. The primary difference between an ESEM and a CFA is that in the former, all cross-loadings are estimated rather than fixed to 0. With these factor analytic specifications^1^, Joshanloo (2016a) found support for three lenses of wellbeing: subjective, psychological, and social, that correlate between 0.30 and 0.60 with each other. Joshanloo and colleagues have replicated this structure of wellbeing across five countries (Joshanloo, 2016b; Joshanloo and Lamers, 2016; Joshanloo et al., 2017a,b; Joshanloo and Jovanovic, 2017). However, these results do not generate factors that map directly onto the original definitions of subjective, psychological, and social wellbeing [see Lamers et al. (2011) for an exception]. For example, self-acceptance and environmental mastery load more on subjective wellbeing than psychological wellbeing, and social integration and social contribution load more on psychological wellbeing than social wellbeing—raising questions about the distinctions between factors (Joshanloo, 2016a). An important consideration with ESEM when cross-loadings are non-negligible is whether the meaning of the factors are different than CFA. As psychometric research on lenses of wellbeing continues, it will be important for scientists to be receptive toward wellbeing lenses that do not map onto popular measurement models or a priori hypotheses. For example, perhaps a major lens of wellbeing should include life satisfaction, positive affect, (infrequent) negative affect, self-acceptance, and environmental mastery (Joshanloo, 2016a).

One lens of wellbeing that is difficult to untangle is eudaimonic wellbeing that includes a combination of psychological and social wellbeing. Some researchers have argued that the six dimensions of psychological wellbeing and the five dimensions of social wellbeing combine to capture Aristotle's philosophical concept of eudaimonia; this approach distinguishes from hedonic pleasures (e.g., momentary positive emotions) and is inclusive of multiple proposed components of wellbeing (Fredrickson et al., 2013). Eudaimonia has been defined in numerous ways by philosophers and psychologists, but the most common definitions often involve living in accordance with one's true self or up to one's full potential (i.e., self-actualization; Deci and Ryan, 2000). According to reviews of existing operational definitions of eudaimonia, there are at least 63 separate elements across different empirical studies and review papers (e.g., from accomplishment and agency to vitality and volunteering; Huta and Waterman, 2014; Martela and Sheldon, 2019).

Whether eudaimonia is a coherent and useful concept for distinguishing aspects of wellbeing has been debated at length (Biswas-Diener et al., 2009; Delle Fave and Bassi, 2009; Kashdan et al., 2008; Keyes and Annas, 2009; Ryan and Huta, 2009; Waterman, 2008). Yet, if we assume the idea of eudaimonia to be worthwhile, there are still questions about its measurement. Studies have suggested that combining psychological and social wellbeing via factor analysis results in poor model fit and that discriminant validity from subjective wellbeing—sometimes called “hedonic wellbeing” in this context—is weak. For example, CFA of eudaimonic and hedonic wellbeing operationalized in this way resulted in subpar model fit (*N* = 84; χ^2^ = 189.40, df = 76, GFI = 0.78, CFI = 0.87, RMSEA = 0.135; Brown et al., 2014). The correlation between observed scores was 0.79, leaving only 30% unique variance after accounting for reliability (α = 0.92). Instead, EFAs suggested a general wellbeing factor and a societal beliefs factor. Direct comparisons of eudaimonic and hedonic wellbeing factors vs. subjective, psychological, and social wellbeing factors suggest separating psychological and social wellbeing is more consistent with the data (Franken et al., 2018; Gallagher et al., 2009). It is worthwhile to note that conceptualizing eudaimonia as a motive/activity, rather than a lens of wellbeing, is predictive of wellbeing and arguably important for understanding the ingredients to the good life (Huta and Waterman, 2014; Steger et al., 2008). As such, many of the elements used in different eudaimonia frameworks are retained in the lower levels of our model.

### Contents of wellbeing

The third level of our hierarchical framework contains contents of wellbeing, which include the topic areas that make up each wellbeing lens. The additional level of contents introduces terms that cut across disciplines within psychological science. Each lens of wellbeing contains two contents: the lens subjective wellbeing includes the contents affect and appraisal; the lens psychological wellbeing includes the contents meaning-making and self-concept; and the lens social wellbeing includes the contents community and interpersonal relationships. Although factor analytic studies have not been conducted on the wellbeing contents proposed, we outline empirical and theoretical work that offers preliminary support for the hierarchy placements.

Over the past four decades, SWB has included both affect and appraisal (Diener, 1984). While the associations between positive affect, (infrequent) negative affect, and life satisfaction are large enough to warrant the higher-order lens of subjective wellbeing, they are also separable (Busseri et al., 2007) with inter-correlations ranging from 0.37 to 0.53 (Busseri, 2018). Research suggests the difference between affect and appraisal in SWB may be in the sources of information used by raters; an individual uses specific events and activities to assess affect, while personal narratives are primarily used for assessing cognitive appraisals (Luhmann et al., 2012). This distinction facilitated research on causal links that connect affect and appraisal. For example, affect was found to mediate self-control and life satisfaction, helping tease apart the wellbeing benefits of self-regulation (Hofmann et al., 2014). Subjective happiness has also become a popular appraisal construct from the SWB tradition whereby participants use their own personal definition of happiness, as opposed to being offered a definition by researchers (Lyubomirsky and Lepper, 1999). There is preliminary evidence that life satisfaction and subjective happiness hang together as appraisal components (Disabato et al., 2016; Vela et al., 2017).

Psychological wellbeing is a heterogeneous model of wellbeing. It is perhaps for this reason that we have yet to see a published conceptual definition of psychological wellbeing. When psychological wellbeing is introduced in scientific articles, the definition tends to be a list of the independent dimensions (that reside at the characteristic level of our hierarchy). As an alternative, we propose the contents of meaning-making and self-concept that allow for more descriptive and coherent groupings of constructs. Of Ryff's (1989) psychological wellbeing dimensions, purpose in life and personal growth are both part of conceptual models of meaning-making (George and Park, 2016), whereas self-acceptance and environmental mastery are part of conceptual models of the self. Existing theories of meaning-making have broadened the number of concepts from purpose in life to include coherence and significance/mattering (Martela and Steger, 2016). Meaning-making includes personal growth, which involves quotidian moments when an individual makes sense of rich experiences (King et al., 2016) along with stressful and even traumatic experiences where felt distress is reframed as growth and often influences a rewriting of mental schemas (Park, 2010).

Of Ryff's (1989) psychological wellbeing dimensions, self-acceptance and environmental mastery directly target an individual's self-concept. Some researchers have noted that self-acceptance and environmental mastery map onto the more commonly used terms “self-esteem” and “self-efficacy” (Burns and Machin, 2009), though Ryff and Singer argue that self-acceptance is distinct from self-esteem (Ryff and Singer, 2008). Using the sociometer definition of self-esteem, that very well might be the case (Leary and Baumeister, 2000). In this model, self-esteem is a belief about how much other people or society view one as valuable and includes perceived social status. From this perspective, Ryff's conceptualization of self-acceptance may be more consistent with the contemporary view of self-compassion, which emphasizes being kind to oneself independent of social value (Bluth and Neff, 2018). Self-efficacy has long been central to an individual's self-concept, with generalized self-efficacy often converging strongly with other measures of self-concept and wellbeing (Judge et al., 2002). Environmental mastery, by definition, is a variant of self-efficacy that involves aspects of locus of control, emphasizing one's internal agency to adapt to an environment (Haidt and Rodin, 1999).

We exclude the dimension of autonomy from our example hierarchy because of its cultural influences. Within the psychological wellbeing framework, autonomy does not mean personal freedom to act how one wishes as it does in Self-Determination Theory (e.g., “I was free to do things my way”; Sheldon and Hilpert, 2012), but rather reflects non-conformity to societal beliefs with a lack of enculturation or convention (e.g., “My decisions are not usually influenced by what everyone else is doing”; Ryff, 1989). While arguably adaptive in Western cultures, it is unclear how applicable this dimension would be in other cultures (Lehman et al., 2004). Indeed, Ryff and Singer (2008) acknowledge “this aspect of wellbeing is undoubtedly the most western of the above dimensions” (p. 23). Future research might explore where the Self-Determination Theory perspective on autonomy might fit into a hierarchical framework of wellbeing given its association with subjective wellbeing is consistent across cultures (Chirkov et al., 2003; Yu et al., 2018).

Keyes' (1998) model of social wellbeing takes a more sociological perspective and captures someone's psychological relationship with their broader communities. Dimensions from his model make up the *community* content in our model. However, Keyes' model of social wellbeing occupies only part of the broader space of social wellbeing, as it leaves out one-on-one social connections that are central to individual's daily lives (e.g., spouses, parents, siblings, friends, co-workers). In our model, we include a separate wellbeing content for interpersonal relationships.

Although there are five dimensions in Keyes' (1998) model, we only include two of the dimensions in our example constructs based on our conceptual definition of wellbeing as being about a person's life and not society. The community content is about one's *relationship* with the communities in which they reside and does not include constructs capturing communities as independent entities. Therefore, the two Keyes' dimensions we include are social integration (the perceived quality of relationships with the local community and broader society) and social contribution (the perceived value one offers as a member of the local community and broader society). The other three dimensions from Keyes' model are excluded because they refer to beliefs about community and society and are not tied to one's self: (1) social acceptance—believing other people in one's community have good qualities such as kindness and trustworthiness, (2) social coherence—understanding what is happening in one's broader society, including viewing it as sensible and predictable, (3) social actualization—believing one's local community and broader society are reaching their potential and will continue to prosper in the future. Keyes draws analogies between social acceptance and self-acceptance, social coherence and meaning in life, and social actualization and personal growth, but factor analytic studies suggest the pairings do not map out empirically. A psychometric analysis of the self-report survey data in Fredrickson et al. suggested that subjective and psychological wellbeing loaded on the same factor together along with social integration and social contribution (Fredrickson et al., 2013), while the other three Keyes' dimensions (social acceptance, social coherence, and social actualization) loaded on their own factor (Brown et al., 2014). A psychometric analysis of the Midlife in the United States—second wave (MIDUS-2) data suggests that only social integration and social contribution loaded on wellbeing factors, while social acceptance, social coherence, and social actualization had weaker associations with other wellbeing dimensions (Disabato, 2018).

The interpersonal relationships content is distinct from Keyes' model. Research suggests the happiest people have the most intimate and successful interpersonal relationships (Lyubomirsky et al., 2005). Studies find that people who can name several people they are close with and whom they share intimate concerns are more likely to report being happy (Diener and Seligman, 2002). We believe the psychological wellbeing dimension of positive relationships with others makes the most sense as a characteristic under the interpersonal relationships content of social wellbeing. Another key component of interpersonal relationships would be relationship quality with others they are close with. Although romantic relationship satisfaction is one important example of relationship quality (Proulx et al., 2007), it is not the only one. Among adults, having at least two high-quality best-friend relationships was associated with greater wellbeing (Birditt and Antonucci, 2007), while sibling relationship quality is central to youth health (Buist et al., 2013). We include perceived social support (contrasted with received social support) because it is theorized to help maintain wellbeing during times of stress (Schwarzer and Leppin, 1989), was integrated into previous frameworks of social wellbeing (Larson, 1992), and is consistently associated with aspects of wellbeing (Chu et al., 2010).

### Characteristics of wellbeing

The fourth level of our hierarchical framework contains characteristics of wellbeing: discrete, clearly defined features of wellbeing that offer practical value in describing human experience. This is perhaps the level at which most individuals conceptualize wellbeing. Many popular wellbeing measurement models focus on the characteristic level: subjective wellbeing (Diener, 1984), psychological wellbeing (Ryff, 1989), social wellbeing (Keyes, 1998), PERMA (Seligman, 2011), and the 63 different elements used when conceptualizing eudaimonia (e.g., authenticity, energy, engagement, hope, respect, sense of coherence; Martela and Sheldon, 2019), etc. Constructs at this level have clearer definitions that lend themselves well to measurement. For example, two of the most popular wellbeing characteristics are positive affect and meaning in life. Positive affect has been defined as “one's level of pleasurable engagement with the environment” (p. 1020; Watson, 1988), and meaning in life has been defined as “a significance beyond the trivial or momentary, to have a purpose, or to have a coherence that transcends chaos” (p. 180; King et al., 2006). Both definitions are more tangible than those for general or lenses of wellbeing. An analogy can be drawn between wellbeing characteristics and the Big 5 facets in the personality literature— personality traits within each broad domain (e.g., extraversion) are called facets (e.g., sociability).

The presence of general wellbeing and lenses of wellbeing levels at the top of the hierarchy does not imply that specific wellbeing characteristics are identical to one another. Positive affect and meaning in life are clearly different constructs, and there are well-supported theories connecting them. People appear to experience more meaning in life when feeling positive affect during laboratory tasks (King et al., 2006). Positive affect in daily life is a stronger predictor of meaning in life than positive uplifts, negative affect, or negative hassles (Machell et al., 2015). Indeed, correlations between positive affect and meaning in life often range from 0.38 to 0.54 (King et al., 2006). These correlations are about the same as those between different facets of the same Big 5 domains. For example, conscientiousness facets correlated between 0.11 and 0.64 in one study (average *r* = 0.43; MacCann et al., 2009) and between 0.15 and 0.54 in another (Roberts et al., 2004). Although the broad domain of conscientiousness is often used, the specific facets are still conceptually and empirically distinct from each other and conscientiousness itself. The same is true for general wellbeing and characteristics such as positive affect and meaning in life.

It is worth reiterating that the characteristics outlined in the wellbeing content section and provided in Table 1 are *examples*. These characteristics are most commonly studied in a psychometric context, but other positively valenced constructs may fit just as well within a hierarchical framework of wellbeing. For example, one review identified 42 different wellbeing assessments with 56 wellbeing characteristics (Cooke et al., 2016). Another review was more inclusive and identified 99 wellbeing assessments comprising 196 wellbeing characteristics (Linton et al., 2016). Many of these other constructs could fit nicely within a hierarchical framework of wellbeing. Although measurement models of wellbeing are often limited to a certain subset of wellbeing characteristics for practical purposes—Seligman (2011) proposed five; Ryff (1989) argued for six facets; Huppert and So (2013) suggested 10; Diener et al. (2009) identified 12; Longo et al. (2017a) presented 14; and Su et al. (2014) offered 18—they are arguably not the limit of available wellbeing characteristics to be studied.

## The “what” and “how” of wellbeing—what counts?

An interesting dilemma that arises from broad overarching frameworks is identifying the boundary of the construct space. In other words, which constructs “count” as wellbeing and which are not wellbeing at all? Agreement among researchers about what constructs should be included in a hierarchical framework of wellbeing is unlikely (Hernandez et al., 2018). We view this as a strength of wellbeing science, not a weakness. Disagreement breeds creativity, and critical discourse and diversity of thought should be encouraged. In personality psychology there is considerable disagreement on the individual facets that subsume the Big 5 domains (Goldberg, 1999). However, the evidence and agreement on the five broad domains of personality is one of the most monumental achievements of psychological scientists. Therefore, we do not view disagreements about the lower levels of a hierarchy as a reason to dismiss a hierarchical framework.

An overly inclusive approach to defining and conceptualizing wellbeing runs the risk of creating tautologies, where the same constructs are used to measure and predict wellbeing (Kashdan et al., 2008). Tautologies are particularly problematic for studying causes of wellbeing. A critique of the Oxford Happiness Scale noted that many of the survey items ask about theoretical *causes* of happiness (e.g., kindness, sense of humor, aesthetic appreciation) rather than happiness itself (Kashdan, 2004). If self-acceptance is used to measure wellbeing, then self-esteem cannot be tested as a predictor of wellbeing. If meaning in life is used to measure wellbeing, then purpose in life cannot be tested as a cause of wellbeing. This same issue has come up in clinical psychology, where constructs like self-criticism and hopelessness have been conceptualized as both symptoms and predictors of depression (Coyne and Whiffen, 1995).

In any study, care needs to be taken to ensure the same construct is not included as both measures and predictors of wellbeing. Depending on which causes of wellbeing are tested, various aspects of wellbeing may have to be excluded from a study's wellbeing measurement model. Of course, this is less of an issue for studies measuring non-psychological causes of wellbeing. For example, a study testing the effectiveness of physical exercise on wellbeing might include a wide array of wellbeing constructs, whereas a study testing what character strengths predict wellbeing might have to include a narrower group of constructs to prevent tautologies.

One solution to avoid tautologies is to categorize positive psychology constructs as predictors, mediators, and outcomes of wellbeing. For example, the “Engine of Wellbeing” structural model organizes personality traits (e.g., neuroticism) and strengths (e.g., curiosity) as predictors of wellbeing, affects (e.g., positive mood) and evaluations (e.g., life satisfaction) as mediators on the way to wellbeing, and behaviors toward meaningful goals (e.g., achievements, relationships) as outcomes of wellbeing (Jayawickreme et al., 2012). Similarly, “The Eudaimonic Activity” structural model organizes eudaimonic motives (e.g., helping others) and activities (e.g., volunteering) as predictors of wellbeing, satisfaction of psychological needs (e.g., relatedness) as mediators, and subjective wellbeing (i.e., life satisfaction, frequent positive affect, infrequent negative affect) as outcomes of wellbeing (Martela and Sheldon, 2019). The engine of wellbeing and eudaimonic activity model each propose narrow definitions of wellbeing to prevent tautologies.

Another solution to avoiding tautologies is to choose constructs that are separable from causes of wellbeing (Headey and Wearing, 1992; Sheldon, 2016). Constructs that resemble mindsets (e.g., optimism), emotion regulation techniques (e.g., savoring), intervention mechanisms of action (e.g., gratitude), life experiences (e.g., accomplishments), and established personality traits (e.g., neuroticism) should be avoided because they can be construed as content-laden predictors/causes of wellbeing. If anything, one's life or self is the content, but nothing else. Constructs such as life satisfaction, subjective happiness, meaning in life, self-esteem, self-efficacy, vitality, positive affect, and infrequent negative affect do not refer to a particular domain of a person's life and make for excellent “content-free” candidates (Magee and Biesanz, 2019). The “content-free” criterion significantly reduces the number of candidate wellbeing constructs and prevents tautologies by having all the content limited to the psychological predictors of wellbeing (Hagerty et al., 2001; Layard, 2010). Indeed, the psychological wellbeing model is a great roadmap for how to get to wellbeing (i.e., causes) and has informed interventions such as wellbeing therapy (Fava et al., 2017). An exemplar construct within positive psychology is gratitude. No wellbeing researchers (that we are aware of) have claimed that gratitude *is* wellbeing, but rather have argued it can produce wellbeing; thus, efficacious gratitude interventions have been developed and tested (Davis et al., 2016). Labeling “content-laden” constructs like gratitude as wellbeing itself would prevent researchers from exploring gratitude as a mediating pathway on the way to wellbeing.

The point of distinguishing between the “what” and “how” of wellbeing is not to discourage research on a diversity of wellbeing constructs. What a shame it would be if wellbeing researchers only studied life satisfaction (Huppert and So, 2013)! Pioneering wellbeing researchers did a wonderful job expanding wellbeing science to move beyond “either a focus on clinical symptomatology such as depression or on global measures of life satisfaction and happiness” (p. 133; Keyes, 1998). Rather than being reductionistic or simplistic, we aim to encourage critical discourse about separating the causes of wellbeing from wellbeing itself. After all, using “content-free” dimensions is not a panacea to the tautology problem. For example, if the Big 5 domains are tested as predictors of wellbeing, then having frequent positive affect and infrequent negative affect as measures of wellbeing is problematic given that they are central to extraversion and neuroticism, respectively (Hayes and Joseph, 2003; Vittersø and Nilsen, 2002). Ultimately, the problem may need to be solved on a study-by-study basis for the time being. We welcome future theoretical work exploring further solutions to differentiating the “what” from the “how” of wellbeing.

## Compare and contrast with alternative wellbeing definitions

Overarching models of wellbeing have moved away from defining wellbeing as a set of components—whether hierarchically organized or not—and instead focused on wholistic definitions of wellbeing. We agree with the movement away from reified lists of a set number of wellbeing variables (e.g., the five PERMA components), and the movement toward conceptual definitions of wellbeing. For example, recent definitions of wellbeing have focused on a sense of balance, equilibrium, or harmony (Bhugra et al., 2013; Galderisi et al., 2015). Some view this as the equilibrium between resources (e.g., personality traits, social network, finances) and challenges (e.g., life events, changes in roles, property loss; Dodge et al., 2012). Wellbeing involves maintaining a homeostatic balance in the presence of life challenges. Others eschew the idea of maximizing high levels of wellbeing components (e.g., positive affect) or combinations of components (e.g., the six PWB dimensions) to describe the good life and replaces it with an emphasis on a harmonious balance between the various aspects of ones self and life (Delle Fave et al., 2023). These definitions seek to contextualize wellbeing within one's life events, developmental tasks, and cultural influences. While we see value in this approach, our definition of wellbeing aims to offer a way to quantitatively assess wellbeing at any moment in a person's life to track wellbeing across these contextual changes. Yes, it is inevitable that people will experience challenges and difficulties in their life that will strain their psychological resources; however, our goal is to measure the waxing and waning of their wellbeing throughout those contexts. In other words, we see context as the predictors of wellbeing rather than wellbeing itself. Drawing from the theory of homeostasis protected mood (Cummins, 2010), the challenge is the context, the homeostasis is how well someone copes with their context—given their resources—and then the outcome is how one is feeling and functioning is their everyday life. We see wellbeing as that outcome capturing *the experience of personally valued fulfillment within one's life*. Our definition of wellbeing is similar to other models of wellbeing through the emphasis on inner peace, contentment, and satisfaction with one's life (e.g., mature happiness; Wong and Bowers, 2018).

## Cultural considerations

Wellbeing research has included large and diverse samples. The World Database of Happiness has data from participants in 55 countries (Veenhoven et al., 1993). Gallup Organization has heavily invested in global wellbeing measurement (and other “positive” constructs like personal strengths; Helliwell et al., 2015). The International Wellbeing Study was an international collaboration of researchers from around the world that included participants from more than 100 countries (http://www.wellbeingstudy.com). Some efforts to study measures across cultures, such as the Personal Wellbeing Index and Pemberton Happiness Index, have included samples from up to 26 different countries in psychometric validations (Hervás and Vázquez, 2013; Żemojtel-Piotrowska et al., 2017). Unfortunately, these examples appear to be the exception rather than the norm—a closer look at the countries represented in wellbeing science suggests that only a subsection of human culture has been examined.

Most of the scientific evidence for a hierarchical framework of wellbeing has come from samples embedded within Western, Educated, Industrialized, Rich, and Democratic (WEIRD) cultures: North America, Western Europe, Oceania, etc. (Henrich et al., 2010). Yet people from WEIRD countries constitute about 12% of the world population. The psychometric studies have primarily used samples from the United States, Canada, United Kingdom, Australia, New Zealand, Italy, Portugal, and the Netherlands (Chen et al., 2013; Huppert et al., 2009; Joshanloo, 2016a; Petrillo et al., 2015; Longo et al., 2016; Lamers et al., 2011). There have been some exceptions, such as samples from Serbia, Kuwait, and Iran (Joshanloo and Jovanovic, 2017; Joshanloo, 2016b; Lambert et al., 2019). Nonetheless, even when participants are sampled from non-WEIRD countries, they often constitute a minority of the total sample. For example, although 60%−80% of the countries in the International Wellbeing Study were non-WEIRD (i.e., depending on what countries are defined as WEIRD), only 18%−44% of the participants were from non-WEIRD countries (Disabato et al., 2016).

A related issue is whether the samples from non-WEIRD countries are representative or if they constitute a unique slice of the country that has disproportionately been exposed to Western culture, higher education, and/or financial wealth. It is unclear if a hierarchical framework of wellbeing is consistent with data from non-WEIRD samples, particularly those that are not engaged in economic globalization (e.g., indigenous cultures). Too often, psychological scientists are quick to argue for the universality of a theory, perhaps modeling their behavior off the physical sciences. However, the social sciences are arguably more complex than the physical sciences, and universal laws of nature are much more difficult to establish. The Big 5 factor structure has not been consistently replicated in indigenous cultures preserved from the globalization of Western media (Heine and Buchtel, 2009). Lack of universality does not discredit a framework or make it useless; rather, it places appropriate boundaries on it. There are almost a billion people embedded in WEIRD cultures for whom the hierarchical framework of wellbeing may apply and be used to enhance their lives. Future scientific evidence will reveal how universal the framework is and whether and how it applies to non-WEIRD cultures.

## Conclusion

Problems exist in wellbeing science such as confusion between the causes and consequences with the nature of wellbeing itself and the jingle-jangle fallacy—where different constructs are mistakenly treated as the same (jingle) or the same construct is treated as different (jangle). To improve the clarity of work by researchers and practitioners, we believe there is generative value in adopting The Hierarchical Framework of Wellbeing (HiFWB). At the top of the hierarchy is general wellbeing (i.e., “h” factor), which can be viewed similarly to general intelligence “g” or general psychopathology “p” and may be just as important as those two constructs for understanding a person's lifespan development. A hierarchical framework then allows psychological scientists to split wellbeing into more and more fine-grained components from lenses to contents to characteristics, depending on their research question. Lower levels of the hierarchy may be particularly useful for psychological practitioners doing case conceptualization. Researchers studying psychological causes or effects of wellbeing need to pay particular attention to tautologies: constructs used to predict wellbeing cannot be used to measure wellbeing itself. We look forward to future research on all levels of the wellbeing hierarchy, spanning from developing construct definitions, organizing constructs into levels, and developing new measures.

## Footnotes

^1^Most of Joshanloo et al.' ESEM models are mathematically identical to EFA models because they include only one exploratory factor measurement model, no confirmatory factor measurement models, and no structural model paths.
